# Human Lobomycosis Caused by *Paracoccidioides* (*Lacazia*) *loboi*, Panama, 2022

**DOI:** 10.3201/eid2912.231092

**Published:** 2023-12

**Authors:** Jose A. Suárez, Betty Cerrud, Monica Pachar, Luz H. Patiño, Jason Reidy, Amina Chace, Roderick Chen-Camaño, Diógenes Alvarado-Barría, Mohammad Z. Nakadar, Juan D. Ramirez, Alberto Paniz-Mondolfi

**Affiliations:** Universidad Internacional Sek Quito, Quito, Ecuador (J.A. Suárez);; Instituto Conmemorativo Gorgas de Estudios de la Salud, Panama City, Panama (B. Cerrud, R. Chen-Camaño);; Hospital Santo Tomas, Panama City (M. Pachar);; Panama College of Environmental Engineer, Panama City (D. Alvarado-Barría);; Icahn School of Medicine at Mount Sinai, New York, New York, USA (J. Reidy, A. Chace, L. H Patiño, M.Z. Nakadar, J.D. Ramirez, A. Paniz-Mondolfi)

**Keywords:** human lobomycosis, Paracoccidioides (Lacazia) loboi, lacaziosis, fungi, fungal infections, Panama

## Abstract

We report a patient from Panama who had lobomycosis caused by *Paracoccidioides* (*Lacazia*) *loboi. *We used combined clinical-epidemiologic and phylogenetic data, including a new gene sequence dataset on this fungus in Panama, for analysis. Findings contribute useful insights to limited knowledge of this fungal infection in the Mesoamerican Biologic Corridor.

Lobomycosis, also known as lacaziosis, is an exceedingly uncommon chronic fungal infection primarily affecting the skin and subcutaneous tissues (*1*). It was identified in the mid-1930s (*2*). This tropical mycosis is caused by the dimorphic fungus *Paracoccidioides *(*Lacazia*) *loboi* (hereafter *P.* *loboi*; see Conclusions) (*3*), and it affects primarily persons residing in regions of Central and South America, particularly the Amazon rainforest (*1*). Clinically, lobomycosis manifests as slow-growing, nodular lesions that can lead to disfigurement and functional impairment if left untreated (*1*). Because of its rarity, diagnostic challenges, and limited therapeutic options, each case of lobomycosis offers valuable insights into its epidemiologic and ecologic aspects, clinical manifestations, treatment strategies, and overall management.

We report a detailed description of a recent patient from Panama who had lobomycosis. We used phylogenetic characterization, microscopic and ultrastructural analysis, providing further insights to the scarce existing knowledge surrounding this intriguing fungal infection across the Mesoamerican Biologic Corridor.

## The Study

An 87-year-old woman from Capira in western Panama was referred to a hospital in Panama in September 2022 to rule out leishmaniasis versus deep fungal infection. Her condition began 40 years earlier with a hyperpigmented nodular lesion on the left leg that progressed to multiple, nonfluctuant, indurated, and exophytic nodular lesions throughout the affected limb, which later involved the scalp. Initially painless, her lesions did not interfere with daily farming activities, but after the first year, occasional pain, partial ulceration, seropurulent/hemorrhagic discharge, and edema in her left leg occurred. No history of penetrating trauma was reported, but she admitted to walking barefoot during agricultural activities. A biopsy performed 3 years earlier reported sporotrichosis, treated with itraconazole and cryotherapy, yielding poor response and persistent lesions. Sizes of lesions had been reduced. Previous clinical and histopathologic reports were not available.

**Figure 1:**
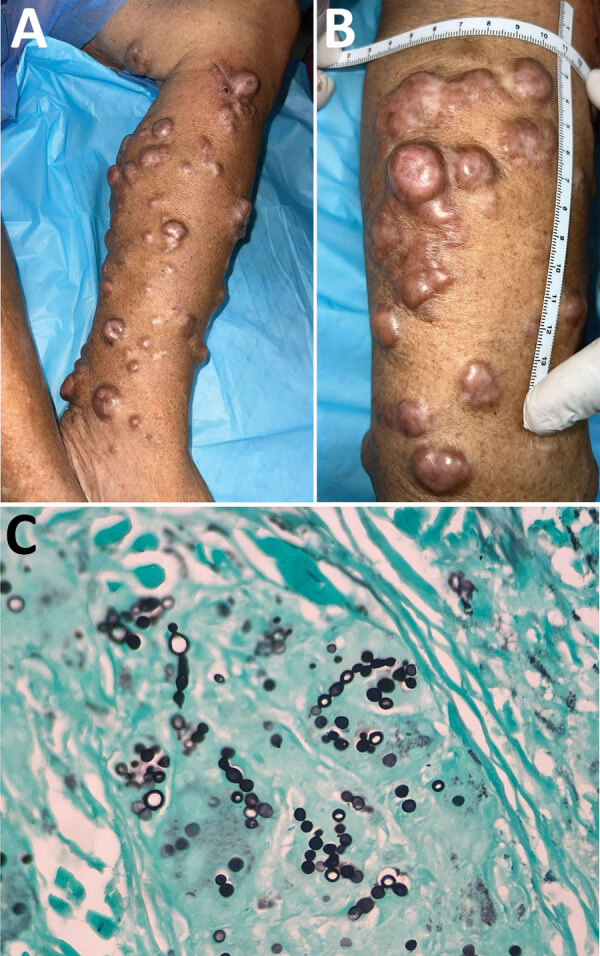
Lesions from an 87-year-old woman from Capira in western Panama, determined to be lobomycosis caused by infection with the fungus *Paracoccidioides* (*Lacazia*) *loboi*. A, B) Multiple keloid-like nodules on the left leg extending through the mid-distal third of the posterior aspect of the left thigh of the patient who had lobomycosis. Shown are the classic confluent arrangement and hyperpigmented aspect of lesions. C) Tissue stain showing classic oval-to-round-shaped cells with connecting tubular projections arranging in a string-of-pearls pattern, both in pairs and individually. Gömöri methenamine silver stain; original magnification ×100.

On physical examination, the patient appeared alert and oriented to person and space. Multiple nodular (keloid-like), confluent, indurated, hyperpigmented, and painless lesions were evident throughout the anterior, medial, lateral, and posterior regions of the left leg; a lesion showing the same characteristics was located in the mid-distal third of the posterior aspect of her left thigh (Figure 1). The patient was tested for leprosy and other cutaneous mycobacterial infections, sporotrichosis, atypical cutaneous leishmaniasis, and neoplasia. The result of a leishmania intradermal reaction test was negative. Direct examination of slit-skin smears for leprosy and leishmania did not show microorganisms. Routine cultures did not grow mycobacteria or fungi. Results of tissue PCR for mycobacteria were negative. However, a biopsy sample submitted for histologic examination showed an extensive chronic granulomatous sclerosing inflammatory reaction, with abundant large and refractile yeast-like structures arranged in chains, consistent with lobomycosis (Figure 1).

**Figure 2:**
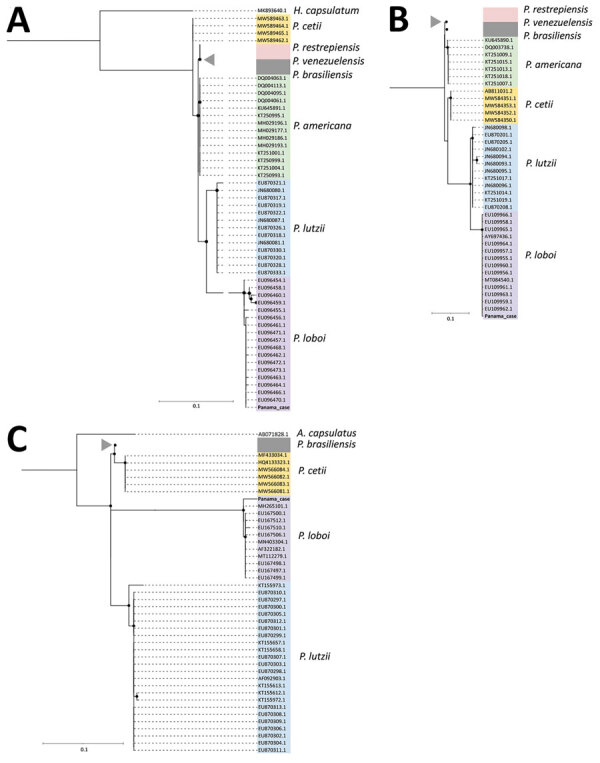
Phylogenetic relationship of *ADP-rf*, *Gp43*, and internal transcribed spacer (ITS) 1 and 2 genes from patient in Panama who had lobomycosis with other *Paracoccidioides* species. Trees indicate phylogenetic analysis inferred by maximum-likelihood method of the *ADP-rf* (A), *Gp43* (B), and ITS1–ITS2 (C) genes. Analyses included sequences obtained from research conducted by Vilela et al. (*7*) and the Panama case analyzed in this study (red). Each color in the trees represent different clusters identified: yellow, *Paracoccidioides cetti*; white, *P. restrepiensis/venezuelensis*; gray, *P. brasiliensis*; green, *P. americana*; light blue, *P. lutzii*; and light purple, *P. loboi*. Gray triangles indicate genomes of the same clade belonging to *P. restrepiensis, P. venezuelensis*, or *P. brasiliensis* (to the *ADP-rf* gene, 49 genomes; to the *Gp43* gene, 32 genomes; and to *ITS1-ITS2* gene, 52 genomes). *Histoplasma capsulatum* and *Ajellomyces capsulatus* DNA sequences were used as outgroups for ADP-rf and ITS genes, respectively. Scale bars indicate nucleotide substitutions per site.

Electron microscopy showed multiple tissue-bound yeast forms exhibiting well-preserved cell walls and classic tubular-filamentous radiations between cells (Appendix Figure 1). We extracted DNA from formalin-fixed, paraffin-embedded tissue and subjected the DNA to PCR for fungal detection. We performed species identification by using the MinION Sequencing System (Oxford Nanopore, https://nanoporetech.com) for *P.* *loboi *ADP-ribosylation factor (ADP-rf), Gp43 protein (Gp43), and internal transcribed spacer 1 and 2 (ITS-1 and ITS-2) genes, as described (*3*,*4*) (Appendix). We evaluated phylogenomic relationships of the isolate sequenced in this study (*5**,**6*) by comparison against other *Paracoccidioides* species, confirming the organism to be *P. loboi* (Figure 2).

In August 2022, the patient was given itraconazole (200 mg orally 1×/d). However, in November 2022, the patient had pyrosis, dyspepsia, and increased liver enzyme levels, leading to the decision to taper her dose of itraconazole to 100 mg/day, resulting in slight improvement of lesions. In June 2023, it was decided to increase the dose of itraconazole to 200 mg/day because of slow improvement of lesions while waiting for adjuvant cryotherapy. Despite showing a slow and minor regression of her nodules, the patient reported a noticeable improvement of dysesthesias associated with the lesions.

## Conclusions

Lobomycosis is a puzzling disease caused by the dimorphic Onygenale fungus *P. loboi* (*8*). One of the intriguing aspects of this organism is its inability to grow in laboratory cultures and its resistance to specific antifungal treatments (*1*). Lobomycosis was documented in the 1930s when Brazilian dermatologist Jorge Lobo described a patient with skin lesions resembling keloids (*2*). Although the fungus is believed to be primarily in soil and vegetation, its presence in marine mammals has been increasingly observed (*1*). Furthermore, human infections have been linked to water proximity, leading to the hypothesis that *P. loboi* might be a hydrophilic microorganism that enters the skin through traumatic means (*9*).

Because of challenges of culturing the fungus in vitro, nomenclature has been a highly debated aspect among mycologists, resulting in diverse classifications of the organism. Proposed taxonomic designations have included *Glenosporella loboi*, *Glenosporopsis amazonica*, *Blastomyces loboi*, *Loboa loboi*, and the family Lobomyces (*1*). In 1999, Taborda et al. proposed a new genus, *Lacazia*, and renamed the agent as *Lacazia loboi* (*10*). Subsequent studies by Mendoza et al. contributed to resolving the taxonomic puzzle surrounding this agent. By conducting amplification of the 18S small subunit rDNA and 600 bp of the chitin synthetase gene, the organism was placed within the group of Onygenales dimorphic fungi (*11*). Moreover, those studies suggested a close phylogenetic relationship between *L. loboi* and *P. brasiliensis*, which was previously inferred on the basis of their morphologic and antigenic similarities (*1*).

More recently, DNA sequence analyses of dolphins samples have shown that the samples align with those of *Paracoccidioides* species (*12*). This discovery has resulted in grouping of this pathogen within a monophyletic cluster, positioned as a close relative to *P. americana* and other *Paracoccidioides* species (*3**,**7*). Consequently, the taxonomy of the dolphin pathogen has been updated, now referred to as *P. cetii*, and the designation of the human-infecting pathogens remains classified as *P. loboi* (*3**,**7**,**12**,**13*). This recent amendment in the taxonomy reflects newfound understanding of the relationships between these pathogens.

Lesions of lobomycosis show various morphologic patterns; 5 well-known subtypes have been identified: infiltrated, keloidal, gummatous, ulcerative, and verrucoid (*1*). Among these subtypes, the keloid subtype is the most common, characterized by well-defined, infiltrative papules and nodules with a shiny, pink surface with overriding telangiectasias (*1*). Nodules are firm to the touch and can merge together. Lobomycosis lesions can appear hyperchromic, hypochromic, or achromic and are typically painless or accompanied by dysesthesia (*1**,**14*). Distinguishing lobomycosis from other infectious causes, such as leishmaniasis, leprosy, sporotrichosis, paracoccidioidomycosis, cryptococcosis, and blastomycosis, can be challenging (*1*). Verrucoid lesions, observed in advanced stages, are commonly found on the legs and show a gray-white coloration mimicking chromoblastomycosis (*1*). Ulcerated lesions resemble those seen in leishmaniasis and mycetomas, further complicating differential diagnosis (*1*).

No antifungal treatment has been consistently effective against *P.* *loboi*. Some drugs have shown partial effectiveness, including amphotericin B, imidazoles, triazoles, expanded spectrum azoles, and clofazimine, but complete remission is rare. Surgical excision remains the most effective option, although multiple or disseminated lesions often recur after surgery (*1*).

Little is known about the ecoepidemiologic and biogeographic aspects of lobomycosis in Panama. This disease was reported in Panama in 1972, followed by 2 additional cases in 1978 (*15*) (Appendix Figure 2). An addendum to the study from 1978 reports 11 additional cases diagnosed and reported by a pathologist in the Chiriquí region (in northern Panama close to the border with Costa Rica) (*15*). Our case-patient came from Capira, which is ≈386 km from Chiriqui in southwestern Panama, suggesting that lobomycosis is endemic to Panama, might be more widespread than previously suspected, and might be underreported (Appendix Figure 2). Increased awareness and further research are needed to better understand the endemicity of this fungal disease in Panama.

Appendix Additional information on human lobomycosis caused by *Paracoccidioides* (*Lacazia*) *loboi*, Panama, 2022.
