# Supplementary material for: Human Lobomycosis Caused by Paracoccidioides (Lacazia) loboi, Panama, 2022
Source: Emerg Infect Dis. 2023 Dec;29(12):2513–7. doi: 10.3201/eid2912.231092 (PMC10683804; doi:10.3201/eid2912.231092)
Supplement: Appendix — Additional information on human lobomycosis caused by Paracoccidioides (Lacazia) loboi, Panama, 2022. [file 23-1092-Techapp-s1.pdf]

Article DOI: https://doi.org/10.3201/eid2912.231092

EID cannot ensure accessibility for supplementary materials supplied by authors. Readers who have difficulty accessing supplementary content should contact the authors for assistance.

Human Lobomycosis Caused by *Paracoccidioides* (*Lacazia*) *loboi*, Panama, 2022

Appendix

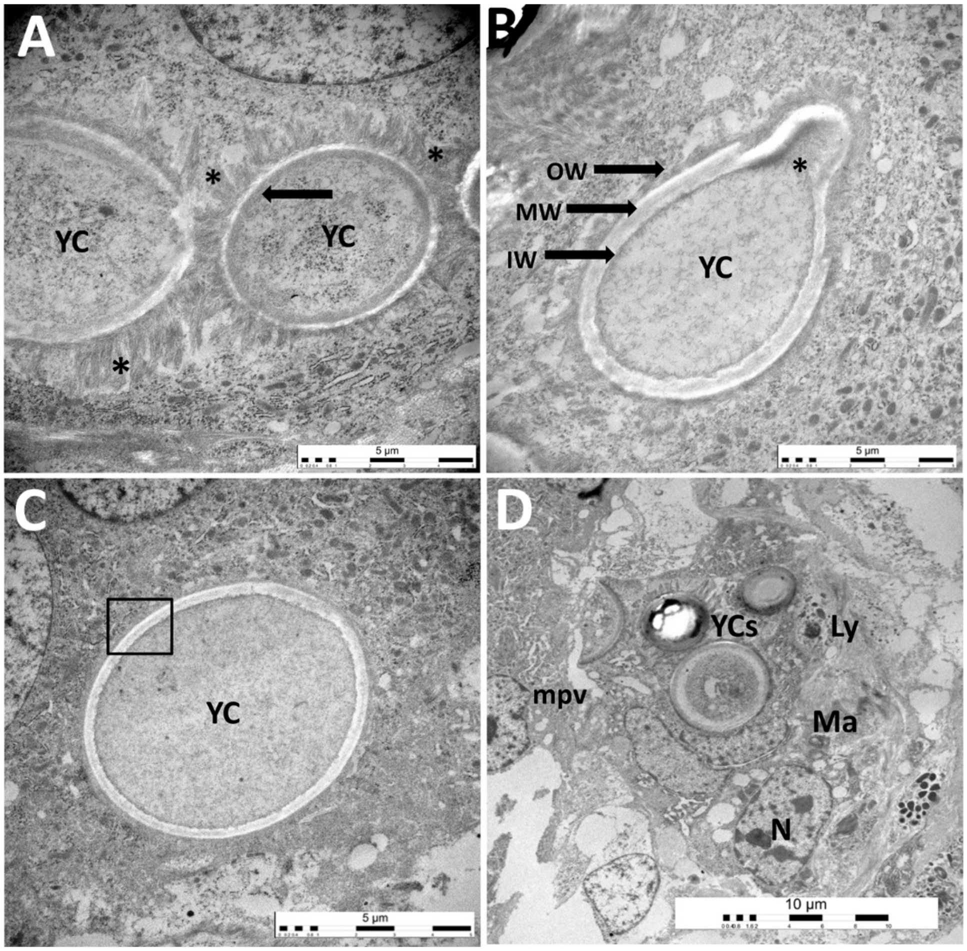

Appendix Figure 1. Transmission electron micrograph depicting ultrastructural features of *Paraccocidioides (Lacazia) loboi.* A) Adjacent yeast cells (YCs) showing characteristic electron-dense

surrounding filamentous radiations (*) projecting outward from the electron-dense cell wall (arrow). Scale bar = 5 μm B) Yeast cell (YC) budding (*) displaying internal (IW) and external (EW) electron-dense aspects of the wall separated by a translucent medial area (MW). Scale bar = 5 μm C) Round yeast cell (YC) highlighting an area the cell wall (enclosed caption) and variably fine electron-dense structures in the cytoplasm. Scale bar = 5 μm D) Multiple yeast cells (YCs) observed within a macrophage (Ma) phagosome showing partially degraded yeast cells (YCs). Ly, lysosomes; mpv, micropinocytotic vesicles; N, nucleus. Scale bar = 10 μm.

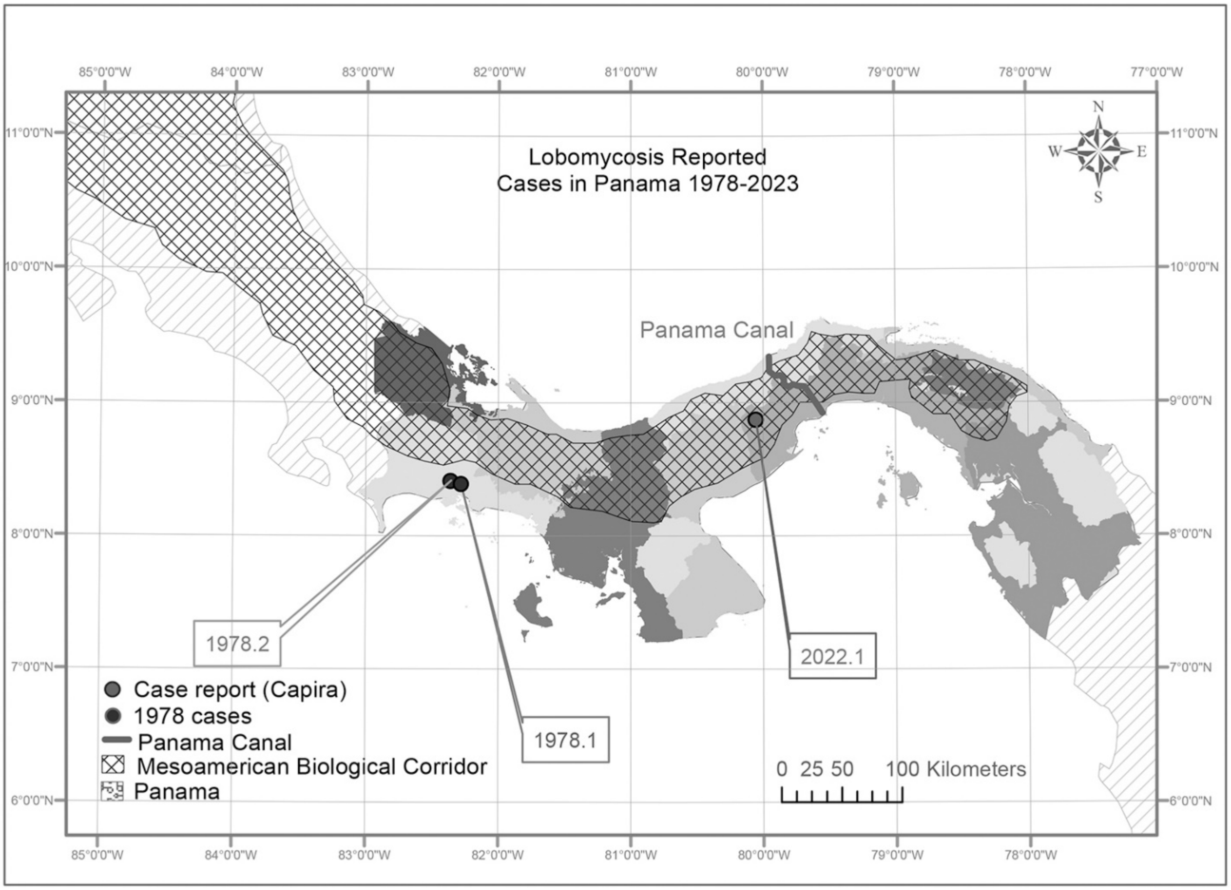

Appendix Figure 2. Geographic occurrence and location of lobomycosis cases in Panama. The current case report (2022.1) originated in the Province of Western Panama, specifically in Ciri Grande County within the Capira District. This location lies in the heart of the Mesoamerican Biologic Corridor, situated to the west of the Panama Canal, at an average elevation of 100 m. In contrast, the 1978 cases (1978.1, 1978.2) were documented more than 45 years ago and occurred more than 350 km from the current case location. Earlier cases were reported in the Chiriqui Province without a specified county location.
